# Mycetoma and the environment

**DOI:** 10.1371/journal.pntd.0011736

**Published:** 2023-11-16

**Authors:** Ahmed Hassan Fahal, Sahar Mubarak Bakhiet

**Affiliations:** The Mycetoma Research Center, University of Khartoum, Khartoum, Sudan; Albert Einstein College of Medicine, UNITED STATES

## Abstract

Mycetoma is a chronic, incapacitating, destructive inflammatory disease with many serious damaging impacts. Currently, there is no control or prevention program as many of its epidemiological characteristics, such as the causative organisms’ ecological niche, natural habitat, primary reservoir, transmission mode, geographical distribution, incidence, and prevalence, remain unclear. This may be due to a lack of research interest, as mycetoma is still a neglected disease and the scarcity of accurate molecular diagnostic techniques in disease-endemic regions for accurate causative microorganisms identification and mapping. With this background, this study set out to address this knowledge gap by considering the mycetoma environmental occurrence predictors. The medical literature obtained data showed a close association between mycetoma occurrence and its environment. The causative microorganisms are available in the environment in active or dormant forms. Animal dung may be a natural niche and reservoir for these organisms, and thorns may facilitate the subcutaneous inoculation. Some environmental factors, such as the soil type and consistency, temperature, water sources, aridity index, and thorny trees, may be risk factors. The population in endemic areas socioeconomic, hygiene, and health education status are contributory factors for mycetoma. The individual’s genetic and immunological backgrounds may determine the disease’s susceptibility and resistance. Environmental conditions and personal hygiene improvement are mandatory to reduce disease occurrence. Mycetoma spatial mapping can detect disease cluster areas and then develop public health strategies for early case detection and management to reduce the disease burden. More research interests and facilities are needed to understand disease pathogenesis and appropriate patient management better.

## Author summary

Mycetoma has been known for centuries, and the earliest report was from the Madras Presidency, India, in 1842 by Gill. Despite that, there is still a knowledge gap in its basic epidemiological characteristics. With this background, this study set out to study the association between mycetoma and its environment to predict its occurrence. All the available medical literature was reviewed to address the study aim. Most of the reports confirmed the presence of the causative organisms of this neglected disease in its surrounding environment. Most are in infective forms, and some are dormant, such as *M*. *mycetomatis*. Certain environmental factors govern the mycetoma occurrence, including the distance from the water source, the thorny tree, the soil type, and others. The animals’ dung may be the natural niche and reservoir for some of these organisms, and sharp objects such as thorns and other skin injury mechanisms may facilitate subcutaneous tissue inoculation. Furthermore, the population’s socioeconomic status and the hygiene standard can contribute to its occurrence. All these predictors should be considered in planning patients’ treatment and disease prevention and control.

## Background

Mycetoma is the most neglected of the neglected tropical diseases; if not managed accurately and appropriately, it will lead to enormous medical, health, and socioeconomic bearings on patients, families, communities, and the health system in the endemic regions [1–3]. It is a chronic, specific, granulomatous, progressively morbid, and disabling subcutaneous inflammatory disease [4–6]. It usually spreads to affect the skin, deep structures, and bones, producing massive tissue destruction, deformities, and disabilities, and hence, it is an important cause of stigma, psychosocial disturbances, and social exclusion of the affected patients [7–9].

More than 70 microorganisms of fungal and bacterial origin were reported to cause mycetoma, and thus, it is classified as eumycetoma and actinomycetoma, respectively [10–12]. These causative agents usually reside in the environment in endemic regions. Their DNA was isolated from the soil, trees, thorns, households, and others [13–15]. Still, how they access the subcutaneous tissue to induce the disease is unclear. The traumatic inoculation of the causative organisms via a minor injury caused by sharp objects is the popular theory. However, many patients deny trauma at the mycetoma site, but it may be a minor trauma that goes unnoticed and forgotten by the patients [16–18]. Furthermore, the mycetoma incubation period is unknown, but it is probably long, and thus, the trauma history may be overlooked [19,20].

**Fig 1:**
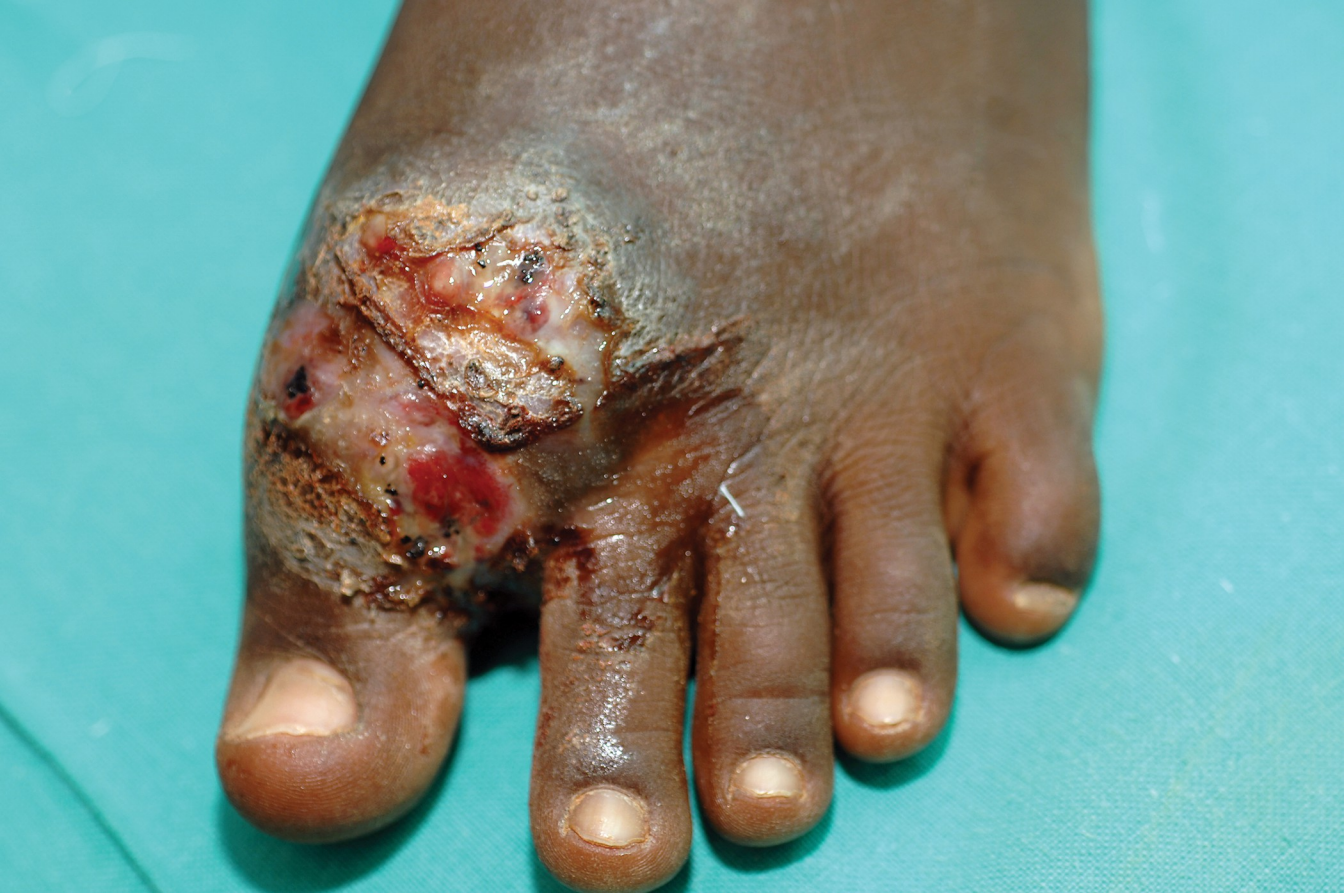
Showing foot eumycetoma with subcutaneous mass, multiple sinuses, and discharge containing black grains.

**Fig 2:**
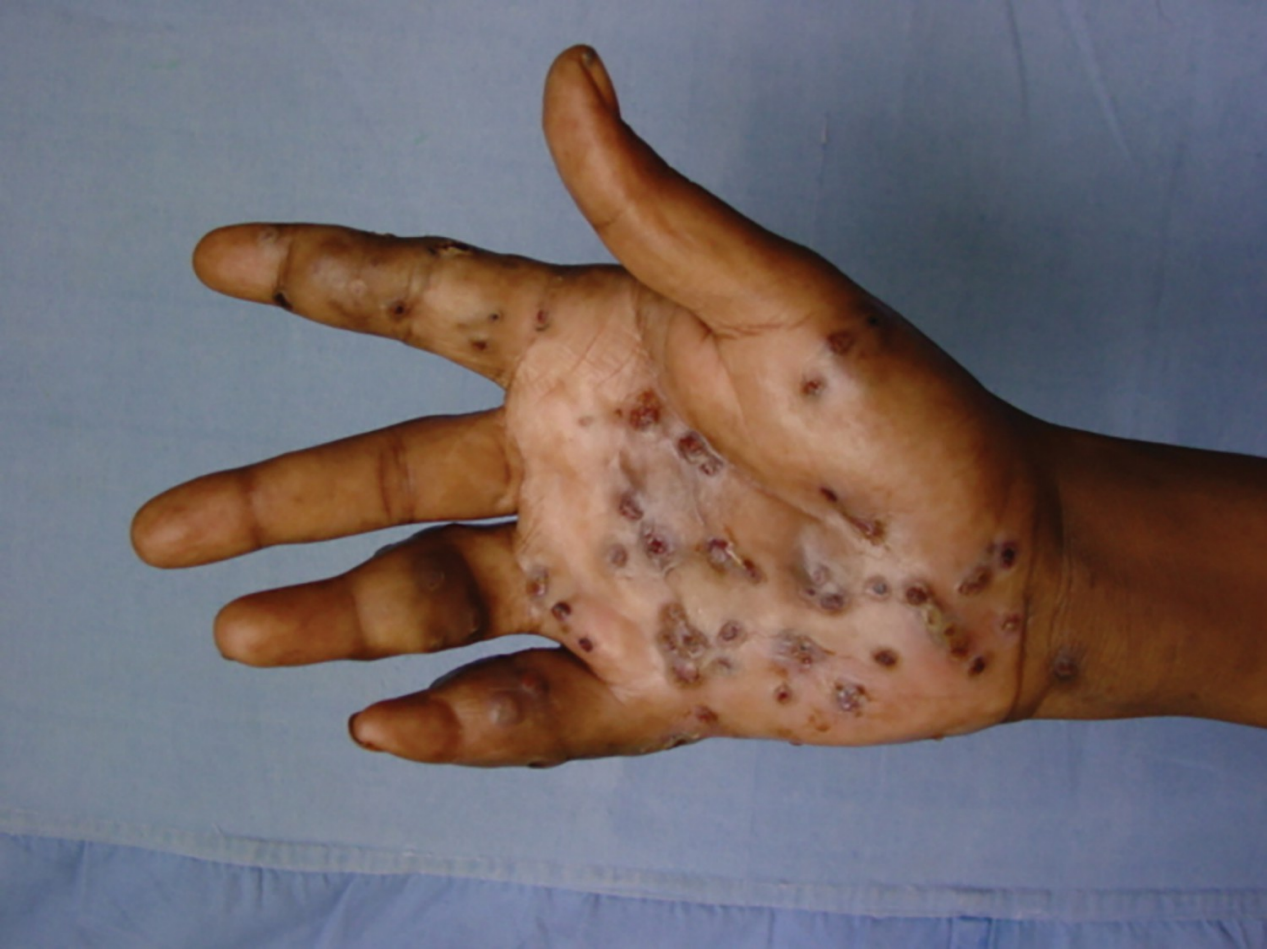
Showing massive hand eumycetoma with multiple masses and multiple sinuses.

The characteristic triad of a painless subcutaneous mass, multiple sinuses, and a discharge containing grains is a mycetoma distinctively characteristic [21,22]. The most common site of mycetoma is the foot (79.2%); the hand ranks as the second most common site (6.6%) [23]. In mycetoma-endemic areas, other body parts may be involved, but less frequently [24–28]. It is clear that mycetoma affects the exposed body parts, and this may support the traumatic subcutaneous inoculation of the environmental mycetoma causative organisms entry route theory (Figs 1 and 2).

### Mycetoma geographical spatial distribution

Mycetoma has an uneven worldwide distribution. It is endemic in tropical and subtropical regions; however, researchers all over the globe have reported its presence [29–31]. It prevails in what is known as the “mycetoma belt,” stretching between the latitudes 15° South and 30° North; however, this is not inclusive as it was reported from 102 countries worldwide [32,33]. The geographical distribution of the individual causative microorganisms is variable [34]. Generally, eumycetoma is frequent in the African continent, and actinomycetoma is more evident in Latin and South America and Asia [35]. However, in the African continent, eumycetoma is more frequent in the eastern part, and actinomycetoma is reported more in the western part [36–39]. Furthermore, there is variable geographical distribution within each contrary. This observation can be explained on an environmental basis, including the rainfall, temperature, and humidity. Mycetoma endemic areas are believed to be characterized by moderate aridity, low humidity, and a short rainy season, but that lacks strong evidence [20,40,41].

Modeling the spatial distribution of mycetoma in Sudan study was conducted at the Mycetoma Research Center (MRC), University of Khartoum, Sudan, to gain more insight into its spatial distribution [42]. The study aimed to identify the mycetoma occurrence environmental predictors and identify areas where any niche predictors are met. Demographic and clinical data of 6,983 mycetoma-confirmed patients seen at the MRC from 1991 to 2018 were included in this study. It included a large occurrence dataset, categorized by type of mycetoma, using a wider set of environmental predictors and a range of modeling algorithms. Regression and machine learning techniques were used to model the relationships between mycetoma occurrence and environmental predictors. In this study, the strongest predictors of mycetoma occurrence were aridity, proximity to water, low soil calcium and sodium concentrations, and the distribution of various thorny trees species.

For eumycetoma occurrence, several environmental predictors were documented and that included the aridity index, soil calcium concentration, wetness index, mean diurnal temperature, distance to the nearest river, presence of cattle, goats and chickens, and the occurrence of *Acacia mellifera* and *Faidherbia albida* trees in the area. However, the distance to the nearest river was the strongest environmental predictor of suitability for eumycetoma occurrence, followed by the diversity of thorny trees, represented by the number of species predicted to be present.

Environmental predictors of actinomycetoma included the aridity index, distance to the nearest river, distance to the nearest water body (pond or lake), wetness index, soil sodium and iron concentrations, presence of cattle and sheep in the area, mean diurnal temperature, and mean temperature in the coldest year quarter. However, the distance to the nearest water body was the major contributor to actinomycetoma occurrence, followed by the distance to the nearest river. Mean temperature during the coldest quarter over a range of 18 to 25°C and the arid areas with a low concentration of soil sodium contributed by almost 13% and 11% occurrence prediction, respectively. The models predicted the occurrence of eumycetoma and actinomycetoma in the central and southeastern states of Sudan and along the Nile River valley and its tributaries.

**Fig 3:**
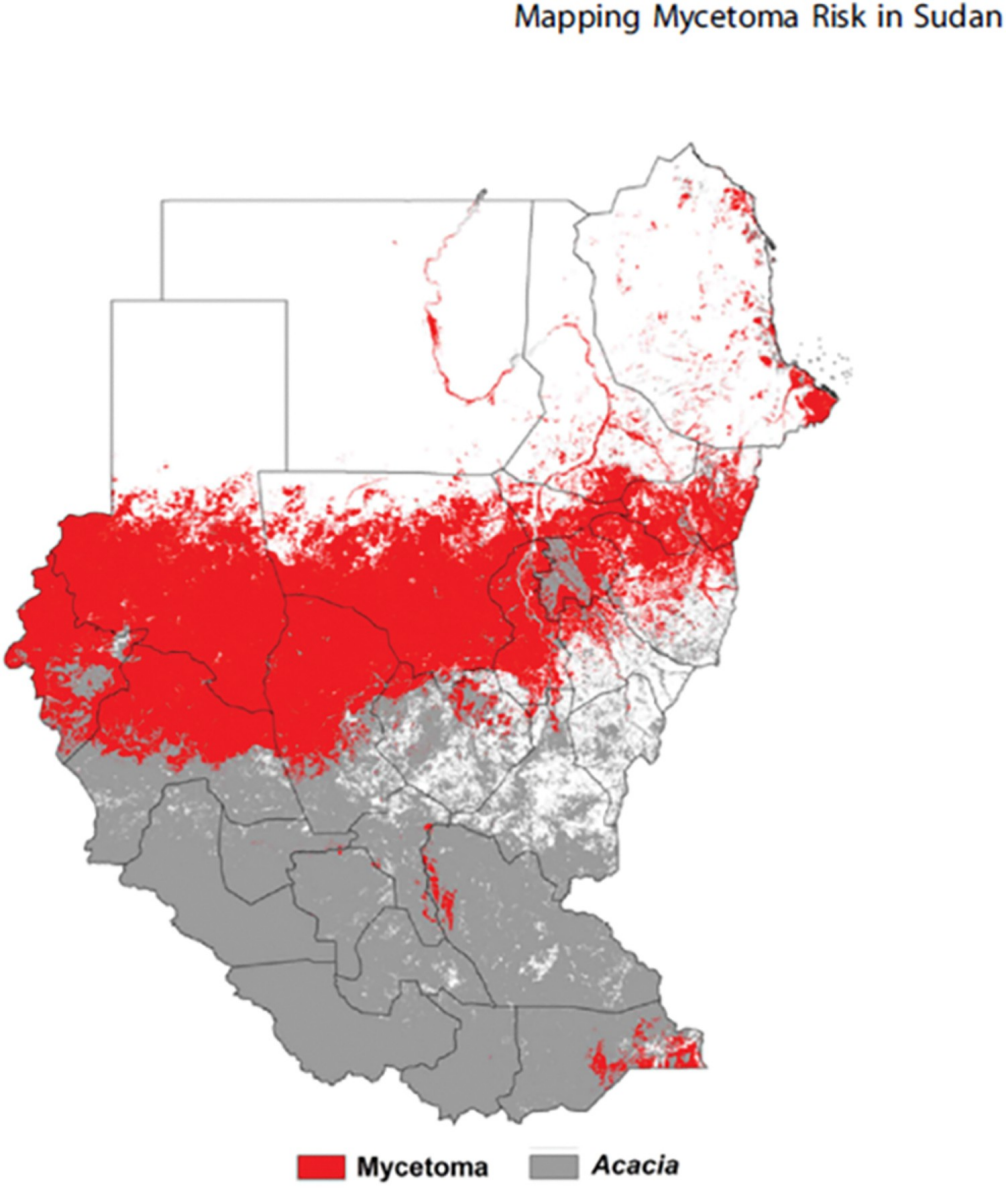
Mapping of the Acacia trees and mycetoma distribution in Sudan and South Sudan. Adopted from Samy AM, van de Sande WWJ, Fahal AH, Peterson AT. Mapping the Potential Risk of Mycetoma Infection in Sudan and South Sudan Using Ecological Niche Modeling. PLoS Negl Trop Dis. 2014; 8[10]: e3250. doi:10.1371/journal.pntd.0003250.

Moreover, another ecological niche modeling (ENM) study was conducted to predict the spatial mycetoma distribution across Sudan and South Sudan [43]. The developed ENMs were based on digital GIS data layers that included the soil characteristics, land surface temperature, and greenness indices to provide a rich picture of environmental variation. The modelings were calibrated in known endemic districts and transferred countrywide. The results suggested that an east-west belt across central Sudan has the greatest mycetoma occurrence risk. The mycetoma and Acacia trees niches were similar, and it also revealed contributions of different environmental factors to mycetoma infection risk but to a lesser extent. However, the main limitation of this study is the small sample size, as only 44 mycetoma patients were included. The data obtained in this study support the soil-borne and Acacia thorn-prick-mediated disease transmission route theory (Fig 3).

Ganawa and his colleagues from Sudan studied the association of mycetoma spatial geographic distribution and environmental predictors in Sennar State, one of the highly endemic regions in the country [44]. They reported an association between mycetoma occurrence and soil type. Most patients (80%) reside in light clay soil, while 13% reside in sandy loam soil. No other environmental factors were significant. In a study from northeast Mexico, the majority of actinomycetoma was reported in areas with kastanozem and lithosol soil types [45]. These reports confirmed the association between mycetoma occurrence and the soil type.

### New causative organisms identification

With the recent development in mycetoma molecular diagnostic techniques, more new causative organisms in different geographical locations were reported. This was impossible in the past as the classical culture technique results were frequently negative or contaminated, as most of these microorganisms are nonsporulating [46]. Many new *Madurella* species were reported, including *Madurella fahalii* from Sudan, *Madurella pseudomycetomatis* from China, and *Madurella tropicana* from Indonesia _[__47__–__49__]._ Other eumycetoma species were identified in Africa and included *Sphaerulina rhododetricola*, *Pleurostomophora ochracea*, *Curvularia pseudolunata*, *Chaetomium atrobrunneum*, *and Microascus gracilis*_[__50__–__53__]._

For actinomycetoma, many new organisms were identified as causative organisms, and these are *Streptomyces sudanensis*, *Actinomadura Mexicana* reported from Sudan, *Actinomadura bengladeshensis* from a patient from Mali, and *Nocardia boironii* from France [54–57].

### Mycetoma and the soil

**Fig 4:**
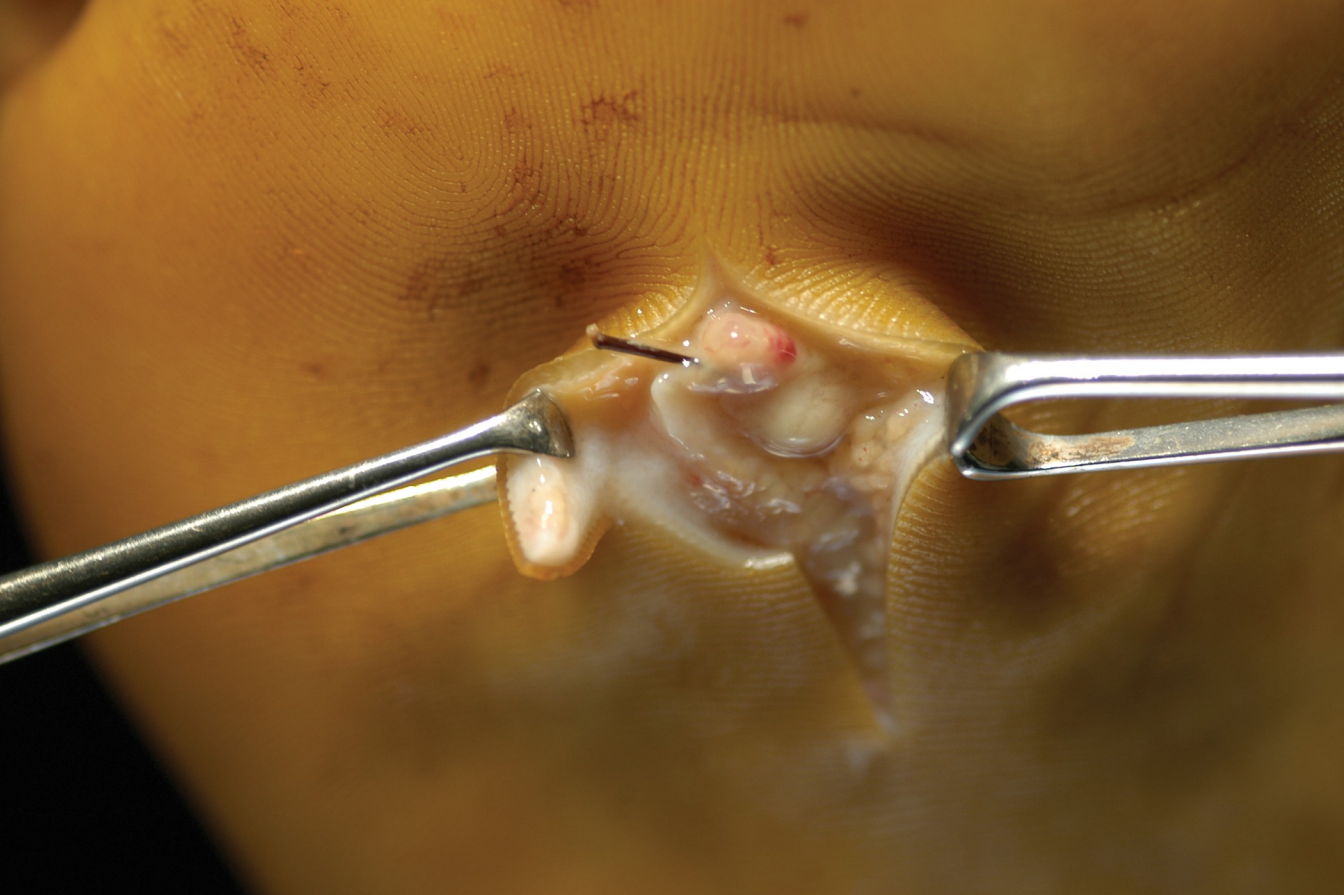
Showing a thorn embedded in the mycetoma granuloma found during surgical excision.

In Sudan, *Madurella mycetomatis* is the main causative agent of human eumycetoma, and it is believed it resides in the soil, and traumatic inoculation is the possible entry route. A field-based study was conducted at the mycetoma endemic regions to verify this [14]. The study included 43 soil samples and 35 thorn collections, and they were cultured to isolate *M*. *mycetomatis*, and ribosomal sequencing was conducted. None of the isolated strains turned out to represent *M*. *mycetomatis* based on either morphological criteria or ribosomal sequencing, and all the obtained black-pigmented fungi belonged to other fungal species. Furthermore, a PCR-RFLP-mediated technique was conducted to identify *M*. *mycetomatis* DNA from the environmental samples and surgical biopsies from patients with mycetoma as control. The results showed that 23% of the soil samples and 5% of the thorns collections had positive PCR, indicating the presence of *M*. *mycetomatis*. Control Dutch and English soil samples were investigated and were negative. These results may support the hypothesis that eumycetoma is primarily environmentally acquired and suggest that *M*. *mycetomatis* needs special conditions for growth, as direct isolation from the environment was not feasible. This hypothesis is supported by the presence of thorns within the mycetoma granuloma during surgery and *M*. *mycetomatis* within thorns in histopathological sections Fig 4. This study is in line with a report from Senegal riverbanks, where *Falcimosporma senegalensis* was cultivated from dry thorns periodically covered by mud during annual floods. *Neotestudina rosati*, an exceptional fungal agent of white-grain mycetoma, was isolated twice from dry, sandy soils [58].

Detection of multiple mycetoma pathogens using fungal metabarcoding analysis of soil DNA in an endemic area of Sudan was reported [13]. Twelve mycetoma causative fungal agents were prevalent, among others: *M*. *mycetomatis*, *M*. *fahalii*, *F*. *senegalensis*, *F*. *tompkinsii*, *Fusarium solani*, and *Curvularia lunata*. These results demonstrate the diffusion of various causative agents in the soils of endemic mycetoma areas and the applicability of fungal metabarcoding analysis in mycetoma geographical mapping.

Another study used a metagenomic approach to study clinical and household environmental samples from 2 different eumycetoma endemic villages in Sudan to determine the eumycetoma-associated fungi and the affected patients habitats association [15]. DNA sequencing targeting the fungal ITS2 domain was performed on soil, animal dung, houses walls and roofs, and Acacia-species thorn samples and compared with culture-dependent methods of fungal isolation. Additionally, the soil samples were compared with those from non-endemic zones. Overall, a total of 392 amplicon sequence variants (ASVs) were detected by ITS2 metagenomics. Eumycetoma causative organisms accounted for 10% of total ASVs which included 11 genera: *Exserohilum* (2%), *Aspergillus* (1.7%), *Curvularia* (1%), *Alternaria* (0.9%), *Madurella* (0.5%), *Fusarium* (0.4%), *Cladosporium* (0.2%), *Exophiala* (0.15%), and, in a lesser extent, *Microascus* (0.05%), *Bipolaris* and *Acremonium* (0.01%) for each. Only 5 genera were identified by culture method, which included *Fusarium* (29%), *Aspergillus* (28%), *Alternaria* (2.5%), *Bipolaris* (1.6%), and *Chaetomium* (0.8%). *M*. *mycetomatis* was detected within all the studied patients’ houses, accounting for 0.7% of total sequences. It was the first commonest eumycetoma-associated agent detected in soil samples and the third common in the dung and house wall samples. In contrast, it was not detected in the house roof, thorn samples, or soils from non-endemic regions. *Exserohilum rostratum*, *Aspergillus* spp., and *Cladosporium* spp. were detected in all samples. *M*. *mycetomatis* and other eumycetoma-associated fungi identified in the patients’ black grains samples by metagenomics were identified in the environmental samples. Only *Acremonium alternatum* and *Falciformispora senegalensis*, responsible for eumycetoma in 2 patients, were not detected, suggesting the infections in these patients happened outside these endemic areas. The soil, animal dung, and houses built from the same soil and dung are the main risk factors for *M*. *mycetomatis* infection in these endemic villages. The study results suggest that poor hygienic and environmental conditions, walking barefooted, and the presence of animals and their dung within the houses may increase eumycetoma risk. Furthermore, the thorns may act as a transmission vector.

### Mycetoma and animals contact

Many studies documented the correlation between mycetoma prevalence and poor environmental conditions, poor personal hygiene, and direct contact with animals and their dung, and where thorns, dirt, and mud prevail [59,60]. It is believed these conditions and others may contribute to this infection by providing a suitable environment for the causative organisms to survive [61]. There is now evidence from phylogenetic studies that *M*. *mycetomatis*, which is nested within the Chaetamiaceae family, is closely related to animals’ dung, and it was suggested that this is the natural niche and reservoir of this organism [62]. In India, *Nocardia asteroids*, a common actinomycetoma causative agent, was also isolated from cow dung [63].

Such poor environmental conditions also are suitable milieu for arthropods vectors to flourish. Ticks were reported in many communities with low hygienic standards [64]. Ticks are second only to mosquitoes as vectors of human infectious diseases worldwide as they are known disease vectors for various diseases of protozoal, rickettsial, spirochetes, viral, fungal, and bacterial origin, and most of these diseases are of zoonotic origin [65]. A study from a mycetoma-endemic village in Sudan reported, for the first time, PCR detection of *Madurella mycetomatis* in ticks that may indicate their possible role in the disease transmission [66]. In Sudan, ticks and tick-borne diseases are widespread, in the studied village, ticks highly infest the animals, and the villagers are in direct contact with these animals. All these observations may suggest an association between mycetoma transmission, animals dung, and ticks. However, the transmission mechanism is unclear. It can be postulated that the tick bites may cause minor injuries that facilitate the inoculation of the organisms into the subcutaneous tissue, or they, in particular, the small immature ticks, actually pass into that tissue, carrying with them the microorganisms.

**Fig 5:**
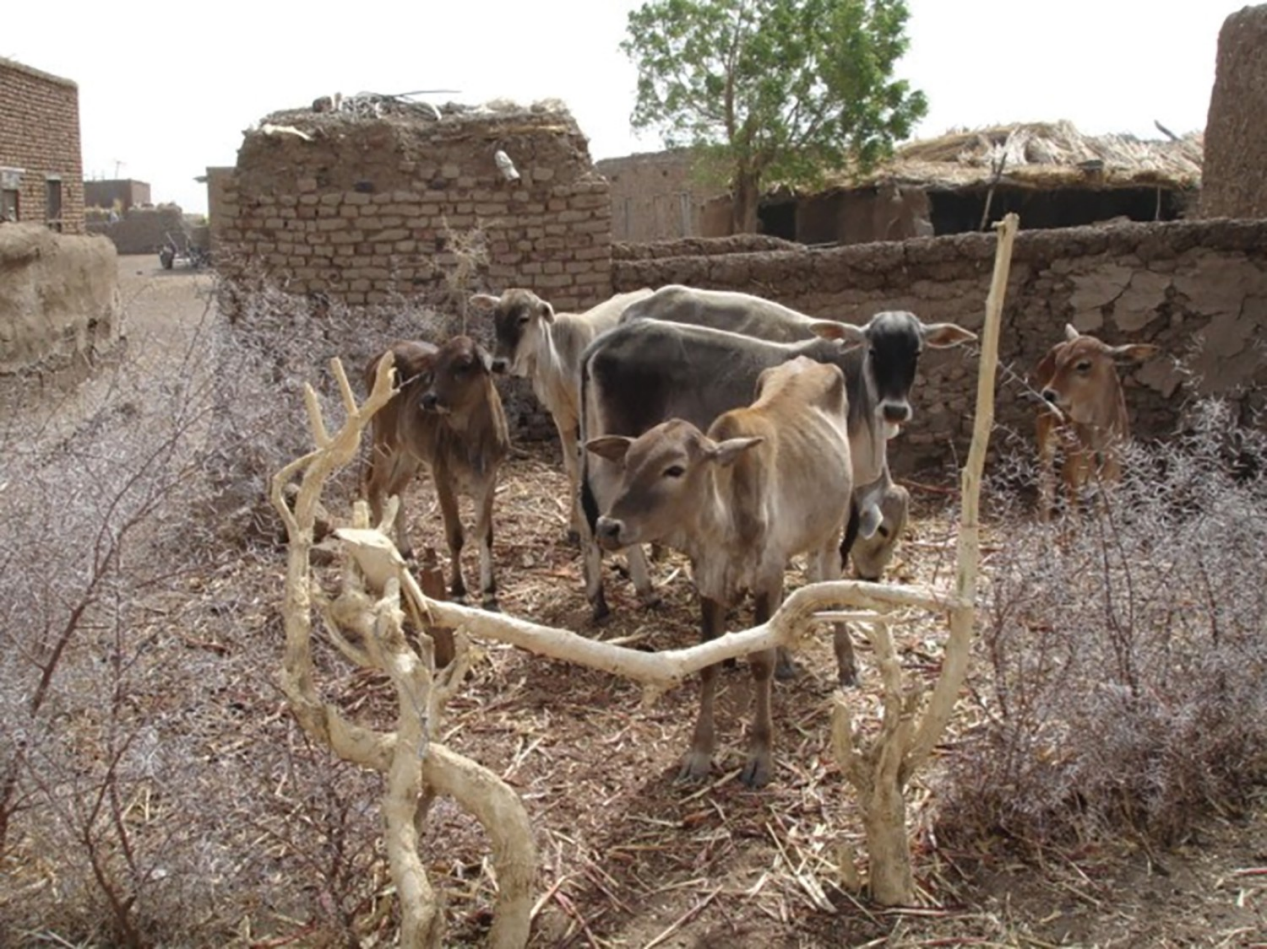
Animal enclosure made of thorns in an endemic region.

Improving the living and hygienic standards in mycetoma endemic regions and villages is essential, and the local villagers should take active roles in this. To reduce the continuous contact with the animals and their dung and the thorny enclosures, new modern hygienic animal cages should be constructed, and villagers should be encouraged to use them (Fig 5, [67]).

### Mycetoma patients’ socioeconomic characteristics

**Fig 6:**
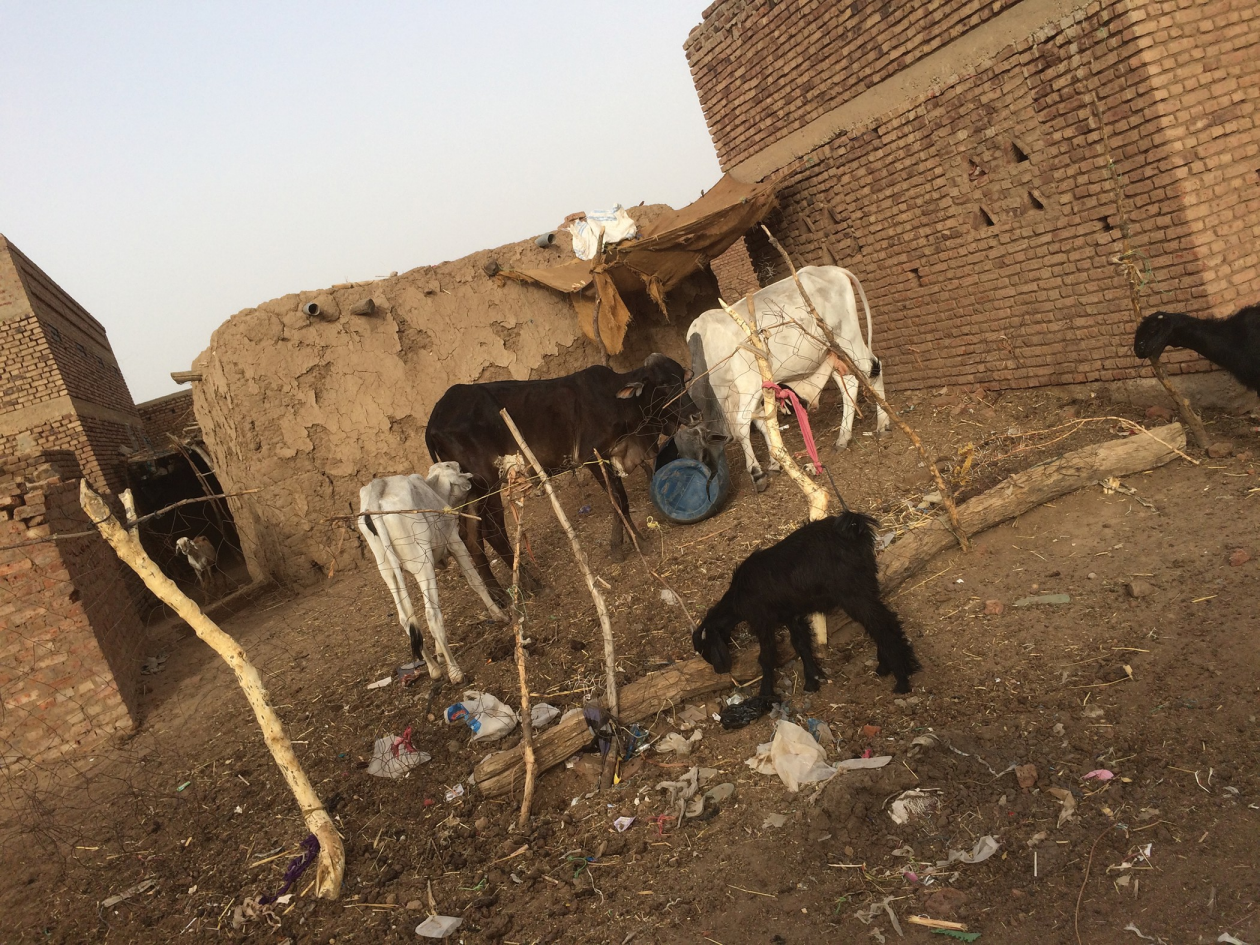
Showing the poor hygienic conditions in a mycetoma endemic village.

Mycetoma is frequently reported among the poorest of the poor in poor, remote communities [68]. A field survey was conducted in Sennar State, Sudan, a highly mycetoma endemic region, to determine the socioeconomic characteristics of the affected patients. It included 7,798 households, 41,176 subjects in 60 villages, and 359 cases of mycetoma were confirmed. Approximately 15% of households with mycetoma had more than 1 household member affected by this disease. Mycetoma households were worse off regarding water supply, toilet facilities, electricity, and electrical appliances than the none affected households. Also, it showed that agricultural practices and reduced access to sanitation and clean water could be risk factors for developing mycetoma. These findings may indicate different environmental factors can contribute to mycetoma development (Fig 6).

### Mycetoma and climate change

There is no evidence to support that climate change in mycetoma endemic or non-endemic regions has affected the mycetoma geographical spatial distribution. One study from Western Rajasthan reported a change in the incidence of actinomycetoma due to climate change [70]. However, it included only 73 mycetoma patients, which is a small study sample size. Furthermore, it attributed the climate change to the increased irrigation by the Rajasthan Canal, which led to rainfall pattern changes, village urbanization, and modification in agricultural practice that converted the desert climate to a humid one, and this is not a true climate change.

A case-control study was conducted in mycetoma endemic villages in Sudan to study the mycetoma individual-level risk factors. It included 359 mycetoma patients and 1,077 healthy sex-matched persons, with no evidence of mycetoma, from the same village as controls. In the study, the odds for mycetoma were almost 3 times higher in individuals in the age group 16 to 30 years [adjusted odds ratio (AOR) = 2.804, 95% CI = 1.424–5.523] compared to those in the age group *≤*15 years. Other factors contributing to the odds of mycetoma were a history of local trauma [AOR = 1.892, 95% CI = 1.425–2.513], being unmarried [AOR = 3.179, 95% CI = 2.339–4.20], and owning livestock [AOR = 3.941, 95% CI = 2.874–5.405]. The obtained results in this study could inform a high index of suspicion for mycetoma diagnosis, which would improve early case detection and management and disease burden reduction [69].

In conclusion, the available literature revealed that mycetoma-causative microorganisms exist in the soil and the environment in endemic regions. Some are dormant as *M*. *mycetomatis*, difficult to isolate by the classical culture method and may become infective in the subcutaneous tissue and cause is an enigma. The animals’ dung may be the natural niche and reservoir for some of these organisms, particularly *M*. *mycetomatis*. Thorns and other skin injury mechanisms may facilitate subcutaneous infection inoculation. The published data showed that mycetoma occurrence is governed by certain environmental predictors such as the distance from the water source, the thorny tree, the soil type, and others. Hence, preventive measures should include environmental and hygiene conditions improvement, using appropriate footwear, reducing contact with animals and their dung, avoiding using thorny animal enclosures, removing animals’ cages outside the endemic villages, and avoiding using the dung as a building material. Sophisticated laboratory techniques are needed in endemic regions to accurately identify and map the environmental mycetoma causative organisms [71]. Furthermore, field surveys and suitability maps should be generated to guide health authorities, academic institutes, and organizations involved in planning national scale surveys for early case detection and management, leading to better patient treatment, prevention, and control of mycetoma.
